# Minimization of Geometric Uncertainties and Setup Errors Improves Tumor Control for Intracranial Stereotactic Radiosurgery

**DOI:** 10.1016/j.adro.2026.102047

**Published:** 2026-03-31

**Authors:** Tenyoh Suzuki, Hidenobu Tachibana, Yuta Takahashi, Kazuya Seki, Weishan Chang

**Affiliations:** a Section of Radiation Safety and Quality Assurance, National Cancer Center Hospital East, Chiba, Japan; b Department of Radiological Sciences, Tokyo Metropolitan University, Tokyo, Japan; c Division of Medical Physics, Jichi Medical University Saitama Medical Center, Saitama, Japan; d Section of Radiation Safety and Quality Assurance, National Cancer Center Hospital, Tokyo, Japan

## Abstract

**Purpose:**

To assess the lesion-level effect of 1-2–mm geometric uncertainty on tumor control probability (TCP) in brain-metastasis stereotactic radiosurgery (SRS), assuming a uniform 2-mm planning target volume margin.

**Methods and Materials:**

We retrospectively analyzed 75 brain metastases in 50 patients who underwent linear accelerator-based SRS. Geometric uncertainty was simulated by applying positional shifts to the treatment plan, and the resulting reduction in TCP was quantified. The resulting dosimetric and radiobiological characteristics were compared between tumors ≤20 mm and those >20 mm.

**Results:**

Tumor size modulated the impact of geometric uncertainty. Small lesions (≤20 mm) exhibited steeper dose gradients (gradient index, 3.6 vs 2.6; *P* = .001) and therefore larger reductions in gross tumor volume D100% under a 2-mm shift (median, 4.5 Gy vs 3.3 Gy; *P* < .001). However, because their initial TCP was high (median 96.6%), the TCP decrement remained modest (median 3.4%). In contrast, large lesions (>20 mm) began with a lower TCP (median 82.1%) and lay on the steep portion of the dose-response curve, so similar or smaller dose losses produced a substantially greater TCP decline (median 10.9% at 2 mm; *P* < .001). The same pattern was observed with 1-mm shifts (ΔTCP 3.5% vs 1.0%).

**Conclusions:**

Patients with larger tumors (>20 mm) may be most vulnerable to a significant loss of tumor control from geometric uncertainties in SRS. This analysis shows promise for personalizing treatment delivery, supporting a shift from applying fixed planning target volume margins toward strategies such as fractionated radiation therapy tailored to individual tumor size and radiobiological characteristics. This hypothesis-generating study requires external validation, with fractionated stereotactic radiation therapy being a subject for future investigation.

## Introduction

Stereotactic radiosurgery (SRS) using a medical linear accelerator (linac) has largely replaced whole-brain radiation therapy as a treatment for multiple brain metastases.^1^^,^^2^ The treatment plans for SRS are designed to increase the maximum dose within the planning target volume (PTV) to 130%-150% of the prescription dose.^3^^,^^4^ This results in an inhomogeneous dose distribution, with dose escalation to the gross tumor volume (GTV) and dose sparing for organs at risk (OARs). However, although this dose escalation contributes to improved local control (LC),^5^ the resulting steep dose gradients make the treatment outcome highly susceptible to geometric uncertainties such as patient setup errors and linac delivery inaccuracies, more so than treatments employing homogeneous dose distributions.

To address this limitation and mitigate the impact of geometric uncertainties, several methods have been proposed for optimizing PTV margins for intracranial SRS. Zhang et al^6^^,^^7^ developed margin formulas specifically for single-fraction treatments, recognizing that the conventional multifraction formulas proposed by van Herk et al^8^ cannot be directly applied because of the absence of error averaging across fractions. Their approach incorporates systematic errors with nonzero mean values to ensure 95% probability of prescribed dose delivery to the clinical target volume in 95% of patients. Subsequently, Takahashi et al^9^ enhanced this methodology by incorporating empirically measured 3-dimensional linac delivery accuracy from multi-institutional star-shot analyses, enabling institution-specific margin calculations based on actual geometric performance.

However, although these approaches can optimize PTV margins against geometric uncertainties, the specific implications of the inhomogeneous dose distribution in this context have not been fully explored. Unlike treatments with homogeneous PTV doses, the inherent dose escalation and steep gradients in SRS mean that a PTV margin ensuring prescription dose coverage to the GTV does not inherently guarantee delivery of the planned centrally escalated dose when geometric shifts occur.^5^ This challenge in defining a margin that robustly delivers the intended inhomogeneous high-dose region to the GTV suggests that geometric uncertainties could lead to variability in the actual GTV dose received, potentially impacting the anticipated LC benefits derived from dose escalation.

In this study, to evaluate the impact of geometric uncertainties on dose reduction and tumor control probability (TCP) while considering the influence of dose inhomogeneity, we performed a retrospective analysis of patients who underwent intracranial SRS.

## Methods and Materials

### Patients and treatment

Fifty consecutive patients with brain metastases who underwent SRS between April 2022 and February 2024 were included in this retrospective study. This study was approved by the institutional review board of National Cancer Center Hospital East (approval number 2017-440). The patient and tumor characteristics are shown in Table 1.

**Table 1:** Patient and tumor characteristics

Number of patients	50
Number of lesions	75
Lesions per patient	
1 lesion	33
2 lesions	11
3 lesions	5
4 lesions	1

All patients were immobilized using the Encompass SRS immobilization system (Qfix). Computed tomography (CT) images with a 1-mm slice thickness were acquired using an Aquilion ONE (Canon Medical Systems). For target delineation, gadolinium-enhanced T1-weighted magnetic resonance imaging (MRI) was also obtained. The CT and MRI were coregistered and transferred to an Eclipse treatment planning system (version 16.1.02, Varian Medical Systems). Target volumes and OARs were delineated by a radiation oncologist. The GTV was contoured on the CT images with reference being made to the MRI. The PTV was generated by creating a 2-mm isotropic margin around the GTV.

Treatment plans were created for volumetric modulated arc therapy with a 10-MV flattening filter-free beam from a TrueBeam linac (Varian Medical Systems). Three or 4 noncoplanar arcs were employed, with isocenter positions automatically determined based on the target’s shape and location. Per our institutional protocol, prescription doses were determined by the attending radiation oncologist: 22 Gy for lesions ≤20 mm and 18 Gy for lesions >20 mm. The plans were normalized such that the prescription isodose line covered 98% of the PTV (*D*_98%_) and optimized so that the dose received by 2% of the PTV (D_2%_) was between 140% and 150% of the prescription dose. The dose constraints for OARs were as follows: cochlea, *D*_max_ < 12 Gy; optic chiasm, *D*_0.2cc_ < 8 Gy and *D*_max_ < 10 Gy; optic nerve, *D*_0.2cc_ < 8 Gy and *D*_max_ < 10 Gy; brainstem, *D*_1cc_ < 10 Gy and *D*_max_ < 15 Gy; spinal cord, *D*_1.2cc_ < 7 Gy, *D*_0.3cc_ < 10 Gy, and *D*_max_ < 14 Gy; and volume of the brain-PTV receiving 12 Gy (*V*_12 Gy_) < 20 cc. Dose calculation was performed using the AcurosXB algorithm with a grid size of 1.25 × 1.25 × 1.0 mm.

### Simulated error

To simulate geometric uncertainties, the isocenter—representing a rigid surrogate for the patient setup position—was shifted independently along the *x, y*, and *z* axes by ±1 mm and ±2 mm from the original treatment plan. Including the original plan, a total of 13 scenarios were performed for each GTV. Combined multidirectional shifts were not applied in this analysis because the aim was to isolate directional effects.

### Evaluation indices

In this study, we used the gradient index (GI) proposed by Paddick et al^10^ to evaluate the dose gradient. GI was calculated using the following equation:

$$\text{GI}=\frac{V_{50\%}}{V_{100\%}},$$

where $V_{50\%}$ is the volume receiving half the prescription dose and $V_{100\%}$ is the prescription isodose volume.

To evaluate LC, we adopted the TCP model parameters from Redmond et al,^11^ which were derived from a pooled analysis of the 1-year LC rates for brain metastases treated with SRS. TCP was calculated based on the physical dose using the following logistic model:

$$\begin{array}{c} TCP={\left(1+{\left(\frac{TD_{50}}{D}\right)}^{4g_{50}}\right)}^{-1}, \end{array}$$

where $TD_{50}$ is the 50% tumor control dose and $g_{50}$ is a parameter describing the steepness of the dose-response curve at $TD_{50}$. Parameter D was defined as the minimum dose covering 100% of the GTV (*D*_100%_). For tumors with a maximum diameter of ≤20 mm, we used $TD_{50}=$ 11.21 Gy and $g_{50}=$ 0.9752. For tumors >20 mm, we used $TD_{50}=$ 13.73 Gy and $g_{50}=$ 0.895. TCP was calculated using the logistic model parameters derived by Redmond et al,^11^ which define the input dose as the prescription dose to a margin. Given that approximately half of the data in the Redmond model were derived from Gamma Knife treatments with 0-mm margins (where margin dose ≈ GTV *D*_100%_), we used GTV *D*_100%_ as the input parameter *D*.

The reductions in both the GTV *D*_100%_ and TCP were calculated. ΔGTV *D*_100%_ and ΔTCP were defined, respectively, as the difference between the GTV *D*_100%_ and TCP values for the original treatment plan and the corresponding values from the plan with the simulated geometric shift.

### Statistical analysis

To compare continuous variables between the 2 groups, the normality of the data was first evaluated using the Shapiro-Wilk test. Welch’s *t* test was then applied for normally distributed data, whereas the Mann-Whitney *U* test was used for non-normally distributed data. To evaluate the directional dependence of positional shifts, differences between the 3 directions (*x, y*, and *z*) were analyzed using the Friedman test. All statistical tests were performed using EZR software version 4.3.1 (Jichi Medical University),^12^ with significance set at *P* < .05 for 2-sided tests.

## Results

Table 2 summarizes the characteristics of the initial treatment plans for 2 groups divided on the basis of tumor size. Of the 75 lesions, 54 had a maximum diameter of ≤20 mm and 21 had a maximum diameter of >20 mm. For lesions ≤20 mm, the median GTV *D*_100%_ was 26.5 Gy (interquartile range [IQR], 25.9-27.1 Gy), and the median initial TCP was 96.6% (IQR, 96.3%-96.9%). For lesions >20 mm, the median GTV *D*_100%_ was 21.0 Gy (IQR, 20.7-21.4 Gy), and the median initial TCP was 82.1% (IQR, 81.4%-83.1%).

**Table 2:** Tumor size, number of lesions, and corresponding dosimetric and TCP parameters

		Median (IQR)	
	n	GTV *D*_100%_ (Gy)	TCP (%)
≤20 mm	54	26.5 (25.9-27.1)	96.6 (96.3-96.9)
>20 mm	21	21.0 (20.7-21.4)	82.1 (81.4-83.1)

GTV = gross tumor volume; TCP = tumor control probability.

**Figure 1:**
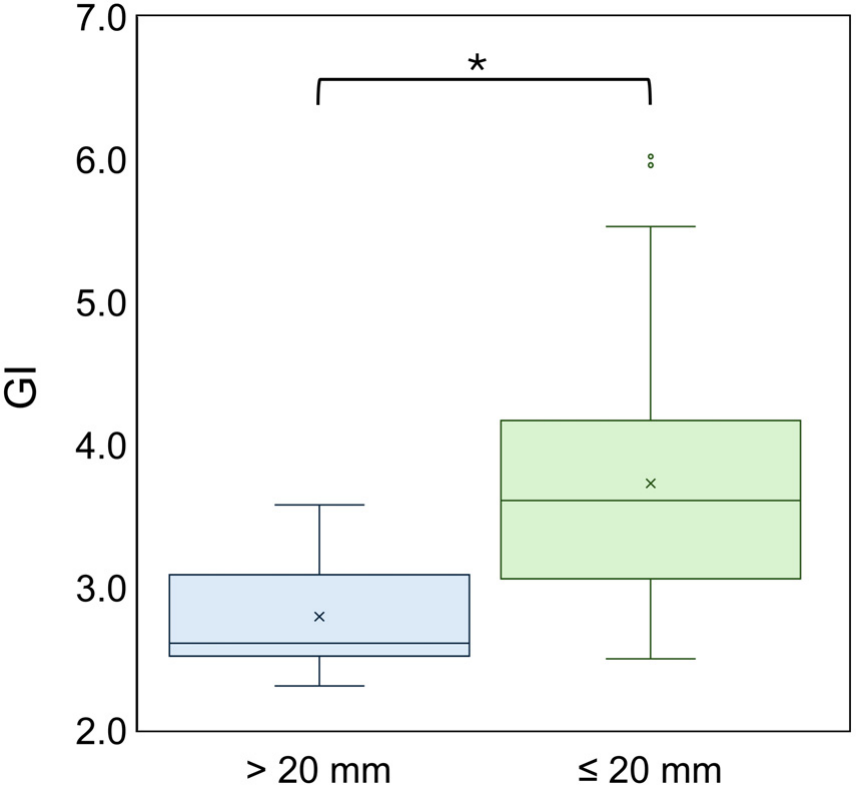
Gradient index (GI) for lesions ≤20 mm (prescription 22 Gy) and >20 mm (prescription 18 Gy). GI was significantly higher in the ≤20 mm group (median 3.6) than in the >20 mm group (median 2.6) (*P* = .001).

Figure 1 shows the GI for the 2 tumor size groups. The median GI was 3.6 (IQR, 2.9-4.0) for lesions ≤20 mm and 2.6 (IQR, 2.5-3.1) for lesions >20 mm and was significantly higher in the ≤20 mm group than in the >20 mm group (*P* = .001).

**Figure 2:**
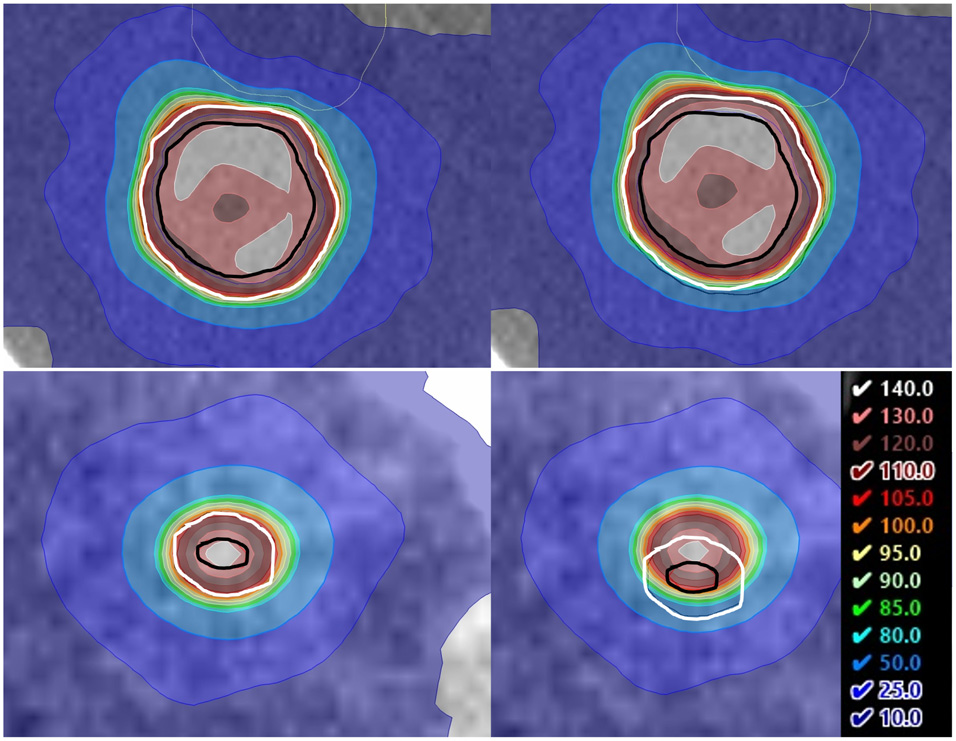
Impact of a 2-mm geometric shift on dose distributions. Two representative cases are shown: a large lesion (>20 mm, top row) and a small lesion (≤20 mm, bottom row). The left and right panels illustrate the unshifted (plan) and shifted (+2 mm) dose distributions, respectively. Black and white contours indicate the gross tumor volume (GTV) and planning target volume (PTV), respectively.

Figure 2 illustrates the impact of a 2-mm geometric shift on tumor dose distribution in 2 representative cases. For the lesions ≤20 mm, the ΔGTV *D*_100%_ was −8.2 Gy (25.5-17.3 Gy), whereas for those >20 mm, the ΔGTV *D*_100%_ was −5.1 Gy (20.7-15.6 Gy). However, the effect on TCP showed an opposite pattern: the smaller lesions showed a reduction of only 11.0 percentage points (from 96% to 85%), whereas the larger lesions showed a reduction of 20 percentage points (from 81% to 61%).

**Figure 3:**
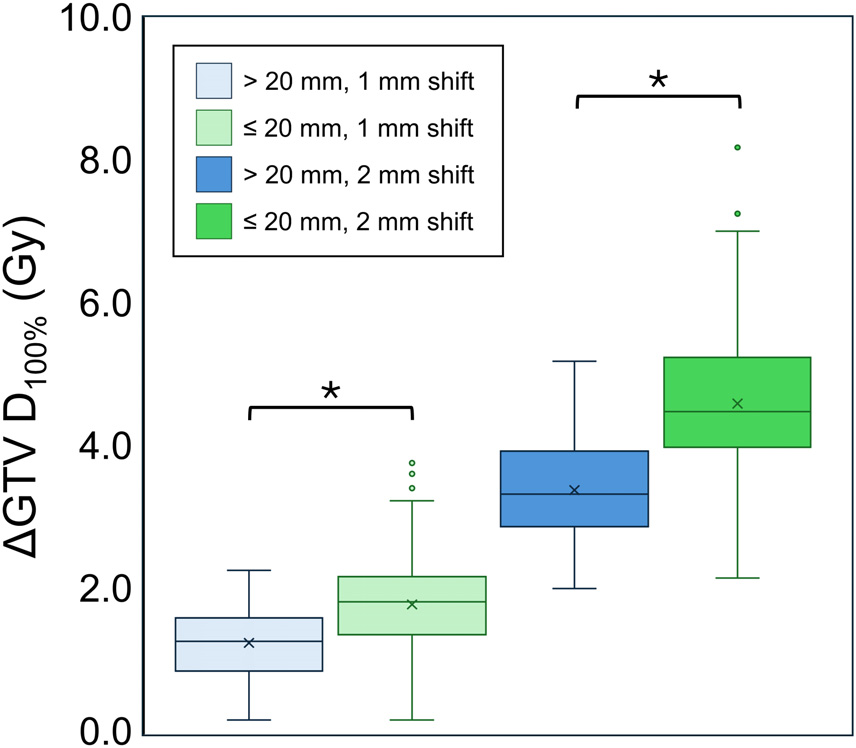
Reduction in the minimum dose covering 100% of the gross tumor volume (GTV) (ΔGTV *D*_100%_) due to simulated geometric shifts. The median ΔGTV D_100%_ was greater in the ≤20 mm group than in the >20mm group for both 1-mm and 2-mm shifts (*P* < .001).

**Figure 4:**
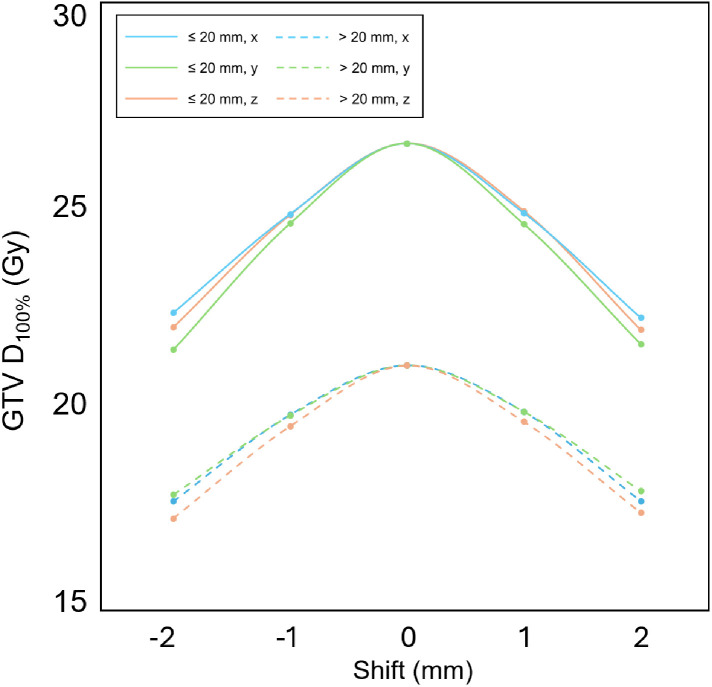
Directional dependence of gross tumor volume (GTV) *D*_100%_ under simulated geometric shifts. The plot shows the GTV *D*_100%_ for translation shifts in the *x* (blue), *y* (green), and *z* (orange) axes. Solid lines represent tumors ≤20 mm and dashed lines represent tumors >20 mm. No significant differences were observed among the 3 shift directions (*P* = .89).

Figure 3 shows the ΔGTV *D*_100%_ due to simulated geometric shifts. For a 1-mm shift, the median ΔGTV D_100%_ was 1.8 Gy (IQR, 1.4-2.2 Gy) in the ≤20 mm group and 1.3 Gy (IQR, 0.9-1.6 Gy) in the >20 mm group. For a 2-mm shift, the median ΔGTV *D*_100%_ was 4.5 Gy (IQR, 4.0-5.3 Gy) in the ≤20 mm group and 3.3 Gy (IQR, 2.9-3.9 Gy) in the >20 mm group. The overall ΔGTV *D*_100%_ was significantly greater in the ≤20 mm group than in the >20 mm group (*P* < .001). Figure 4 illustrates the GTV *D*_100%_ as a function of positional shifts in the *x, y*, and *z* directions. Statistical analysis confirmed that there was no significant directional dependence in dose reduction for either tumor size group (*P* = .89).

**Figure 5:**
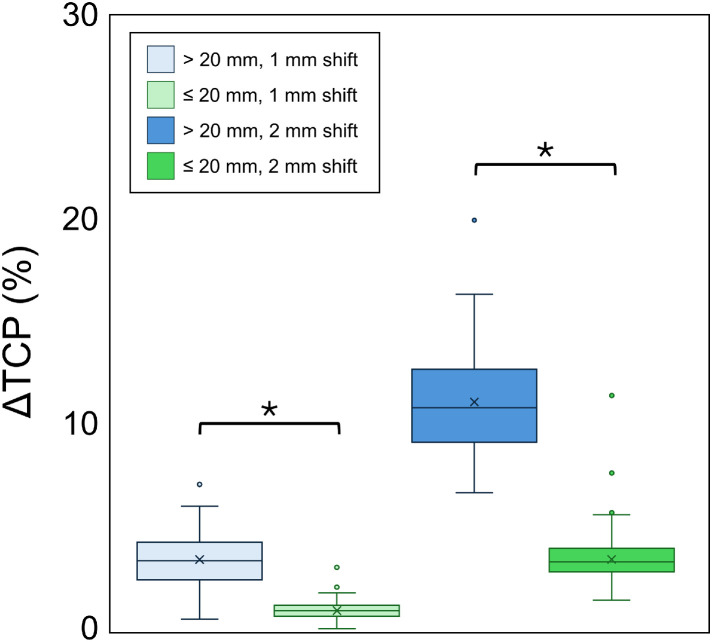
Reduction in tumor control probability (ΔTCP) due to simulated geometric shifts. The median ΔTCP was significantly greater in the >20 mm group than in the ≤20 mm group for both 1-mm and 2-mm shifts (*P* < .001).

Figure 5 shows the ΔTCP resulting from simulated geometric shifts. For a 1-mm shift, the median ΔTCP was 1.0% (IQR, 0.8%-1.3%) in the ≤20 mm group and 3.5% (IQR, 2.5%-4.3%) in the >20 mm group. For a 2-mm shift, the median ΔTCP was 3.4% (IQR, 2.9%-4.1%) in the ≤20 mm group and 10.9% (IQR, 9.3%-12.7%) in the >20 mm group. The ΔTCP was significantly greater in the >20 mm group than in the ≤20 mm group across both shift conditions (*P* < .001).

## Discussion

In this study, we evaluated how geometric uncertainties in SRS for brain metastases affect dose and TCP reduction as a function of tumor size and the corresponding prescription dose. Our results demonstrate that the impact of positional shifts varies with tumor size: smaller tumors (≤20 mm) experience a greater physical dose reduction to the GTV, whereas larger tumors (>20 mm) experience a more pronounced decrease in TCP.

The greater dose reduction observed in smaller tumors can be attributed to the characteristics of their dose distributions. To spare surrounding normal brain tissue, treatment plans for small targets typically employ very steep dose gradients at the periphery of the target. Such steep gradients mean that even minimal positional shifts can cause portions of the GTV to fall outside the high-dose region, resulting in a substantial decrease in GTV *D*_100%_. In contrast, larger tumors generally have shallower dose gradients than smaller tumors, rendering them more robust against positional shifts in terms of GTV dose coverage. Our finding that steeper dose gradients are more sensitive to geometric uncertainties in dose coverage is consistent with the report by Yoon et al.^13^ In steep-gradient SRS dose distributions, minimum-dose–based metrics such as GTV *D*_100%_ may provide complementary information to mean-dose–based metrics when evaluating robustness against geometric uncertainties.

However, the marked reduction in TCP observed for larger tumors is likely explained by the characteristics of the TCP curve. In this study, the large-tumor group was treated with a relatively lower prescription dose (18 Gy), resulting in an initial TCP of approximately 82%. This value lies within the steep “shoulder” region of the TCP curve, where even small dose reductions can cause substantial decreases in TCP. Conversely, smaller tumors treated with higher doses (22 Gy) achieved an initial TCP exceeding 96%, placing them in the “plateau” region of the TCP curve, where TCP is relatively insensitive to dose reductions. As a result, even substantial decreases in GTV dose translated into only modest declines in TCP in the small-tumor group. Some previous studies have reported that smaller tumors are more susceptible to TCP loss from geometric uncertainties,^14^^,^^15^ which at first glance appears inconsistent with our results. However, these studies were based on phantom or mathematical modeling, and TCP was estimated using models incorporating an equivalent uniform dose. In contrast, our study used real clinical data with prescription doses adjusted for tumor size and employed a TCP model derived from actual clinical outcomes. Given these fundamentally different assumptions, we hold that our results are more likely to reflect real-world clinical scenarios.

Our findings have important clinical implications for SRS treatment strategies. Although smaller tumors are more susceptible to dose reductions, their TCP tends to remain high, making them appear robust in terms of clinical outcome. In contrast, larger tumors demonstrated greater vulnerability to geometric uncertainties from a clinical outcome perspective. This suggests that the standard 2-mm PTV margin may be insufficient to guarantee the expected tumor control for large lesions. Although a 2-mm margin may seem adequate given current immobilization technologies and linac delivery accuracy, when Takahashi et al^9^ used gel dosimetry with a 3D star-shot test to assess SRS delivery accuracy in Varian and Elekta linacs, they found that 1 Varian-equipped institution and 9 Elekta-equipped institutions failed to meet the 1-mm targeting accuracy criterion for SRS. Moreover, their calculations indicated that to achieve 95% probability of covering 95% of the tumor volume, a margin of 2.3 mm was required in the cranio-caudal direction for Varian linacs, and 3.5 mm for Elekta linacs.^9^ These results suggest that the currently adopted 2-mm margin may not always be sufficient.^9^ When delivery inaccuracies are considered alongside our findings regarding clinical vulnerability, the necessity for institutional end-to-end verification is further underscored. Such verification is important to ensure that the planned dose is accurately delivered to the target, especially because geometric errors in SRS, particularly for larger tumors, can directly compromise clinical outcomes. The isotropic sensitivity observed across all axes supports the use of uniform PTV margins for treatment systems with symmetrical delivery profiles (Fig. 4), whereas nonuniform expansions may be warranted if institutional end-to-end testing reveals specific directional biases. In cases where sufficient targeting accuracy cannot be guaranteed, increasing the PTV margin can be considered; however, because normal brain tissue lies immediately outside the GTV, increasing the margin also increases the risk of radiation-induced brain necrosis.^16^^,^^17^ If the margin cannot be safely increased, alternative strategies such as fractionated stereotactic radiation therapy (SRT) may present a promising option. Fractionation has the potential to mitigate the impact of geometric uncertainties in any single fraction while also providing radiobiological advantages, and these could contribute to improved treatment robustness.^9^^,^^18^

This study has several limitations. Although it is a retrospective analysis, all eligible patients presenting during the study period were consecutively included, minimizing selection bias. Our simulations considered only hypothetical translational errors of ±1 mm and ±2 mm, without accounting for rotational errors, which may also occur in clinical practice. Our quantitative findings are based on a TCP model parameterized using GTV *D*_100%_ that was originally reported by Redmond et al^11^; therefore, absolute TCP values may differ if alternative dose descriptors are used. However, we expect the qualitative trends observed to remain consistent. It should be acknowledged that the TCP model^11^ is likely to already reflect inherent clinical uncertainties, such as isocenter discrepancies.^9^ Consequently, our simulated shifts may effectively superimpose additional errors on these baseline uncertainties. Nevertheless, because this study focuses on the relative sensitivity between tumor sizes, rather than on absolute TCP values, we believe the qualitative trends remain valid for assessing further clinical risk.

## Conclusions

Geometric uncertainties in SRS exert different impacts on treatment efficacy depending on tumor size and prescription dose. Although small tumors are more prone to physical dose reduction, their TCP tends to remain high. In contrast, large tumors are relatively robust in terms of dose coverage but are more vulnerable to declines in TCP. These findings suggest that while minimizing geometric errors is essential for all cases, special attention should be paid to the clinical impact of such uncertainties on large tumors. Furthermore, fractionated SRT may represent a promising alternative subject for further investigation, particularly in cases where maintaining or increasing PTV margins is infeasible. Our findings suggest the potential for individualized treatment strategies based on tumor size; however, prospective multicenter validation is essential before clinical implementation.

## Disclosures

The authors declare that they have no known competing financial interests or personal relationships that could have appeared to influence the work reported in this paper.
